# Akt Links Insulin Signaling to Albumin Endocytosis in Proximal Tubule Epithelial Cells

**DOI:** 10.1371/journal.pone.0140417

**Published:** 2015-10-14

**Authors:** Sam Coffey, Tina Costacou, Trevor Orchard, Elif Erkan

**Affiliations:** 1 Cincinnati Children’s Hospital Medical Center, Division of Nephrology, Cincinnati, OH, United States of America; 2 University of Pittsburgh, Department of Epidemiology, Pittsburgh, United States of America; INSERM, FRANCE

## Abstract

Diabetes mellitus (DM) has become an epidemic, causing a significant decline in quality of life of individuals due to its multisystem involvement. Kidney is an important target organ in DM accounting for the majority of patients requiring renal replacement therapy at dialysis units. Microalbuminuria (MA) has been a valuable tool to predict end-organ damage in DM but its low sensitivity has driven research efforts to seek other alternatives. Albumin is taken up by albumin receptors, megalin and cubilin in the proximal tubule epithelial cells. We demonstrated that insulin at physiological concentrations induce albumin endocytosis through activation of protein kinase B (Akt) in proximal tubule epithelial cells. Inhibition of Akt by a phosphorylation deficient construct abrogated insulin induced albumin endocytosis suggesting a role for Akt in insulin-induced albumin endocytosis. Furthermore we demonstrated a novel interaction between Akt substrate 160kDa (AS160) and cytoplasmic tail of megalin. Mice with type 1 DM (T1D) displayed decreased Akt, megalin, cubilin and AS160 expression in their kidneys in association with urinary cubilin shedding preceding significant MA. Patients with T1D who have developed MA in the EDC (The Pittsburgh Epidemiology of Diabetes Complications) study demonstrated urinary cubilin shedding prior to development of MA. We hypothesize that perturbed insulin-Akt cascade in DM leads to alterations in trafficking of megalin and cubilin, which results in urinary cubilin shedding as a prelude to MA in early diabetic nephropathy. We propose that utilization of urinary cubilin shedding, as a urinary biomarker, will allow us to detect and intervene in diabetic nephropathy (DN) at an earlier stage.

## Introduction

Kidney is a major target organ in DM accounting for forty five percent of new patients starting on renal replacement therapy [1]. In a recent study, an increase in prevalence of type 1 and type 2 DM by 21.1% and 30.5% respectively, was reported among children and adolescents in US between 2001–2009 [2]. A similar trend was reported in Europe and other countries [3, 4]. Considering the future burden of the increased number of adults with DN, it is imperative to implement screening tools to detect early DN. Microalbuminuria is utilized as a clinical tool to monitor kidney damage and a predictive marker for future cardiovascular and neurocognitive complications [5, 6]. The pathology that underlies MA remains undetermined but is proposed to be multifactorial stemming from hyperfiltration, reactive oxygen species and endothelial dysfunction caused by the diabetic milieu. Previous reports of low sensitivity and advanced renal damage at the time of MA has prompted research efforts seeking alternative biomarkers to capture early molecular alterations in the kidney to predict development of DN [7–9].

The proximal tubule displays an intricate network of endocytic receptors and adaptor proteins to navigate retrieval of albumin, carrier-bound vitamins, trace elements and amino acids. Megalin, cubilin and amnionless are the main receptors responsible for albumin endocytosis in proximal tubule epithelial cells. Megalin and cubilin receptor complex is instrumental in execution of albumin endocytosis in proximal tubule epithelial cells. Megalin and cubilin are colocalized on the brush border, within the coated pits, endocytic vesicles, and recycling endosomes [10–12]. Endocytosis of megalin is promoted by the adaptor protein disabled-2 (Dab2), which binds to the FXNPXY motif in megalin [13–15]. Dab2 knock-out mice display decreased endocytosis, impaired megalin/cubilin trafficking in the visceral endoderm and proteinuria. This mutually dependent presence of cubilin, megalin and Dab2 emphasizes the close coordination between these proteins in order to maintain the fidelity of ligand uptake and sorting. However, how dysregulation of this receptor-adaptor protein complex contributes to development of MA is unknown.

Recently, an association between endocytic proteins and progression of kidney diseases and albuminuria was shown in human genome-wide studies. A missense variant (I2984V) in the cubilin gene CUBN was associated with a 41% increased risk for development of persistent MA during 20 years of follow-up among 1304 participants with type 1 diabetes in a prospective study [16]. Furthermore, a genetic variant of cubilin was identified as a novel risk variant for kidney function loss in end-stage renal disease and graft loss in kidney transplantation [17]. Genome-wide studies have also identified Dab2 as a locus affecting kidney function and susceptibility to chronic kidney disease [18–19]. Taken together these data strongly support the hypothesis that alterations in expressions and interactions between endocytic receptors and adaptor proteins play an important role in development MA and progression of kidney diseases.

We hypothesize that endocytic machinery in proximal tubule epithelial cell is a target for insulin and perturbations of this pathway result in mishandling of albumin in the proximal tubule that eventually leads to MA in DM. We believe that delineating the mechanism of protein-protein interactions surrounding insulin evoked activation of signaling events in the proximal tubule is pivotal in understanding the origins of MA in early stages of DN. We propose that because of its proximity to the urinary space, molecular cues indicating the disarrangements in the cross talk between insulin signaling and endocytic pathway in the proximal tubule may be captured in the urine. Albumin receptors megalin and cubilin both possess long extracytosolic domain floating in the urinary space. Cubilin lacks a transmembrane domain and is dependent on megalin for internalization and recycling. We hypothesize that shedding of cubilin in the urinary space can be considered as a sign for perturbation of the insulin induced albumin endocytosis and serve as a biomarker to predict MA.

We reported that protein kinase B (Akt), a pivotal protein in insulin signaling, mediates albumin endocytosis in proximal tubule epithelial cells through its interaction with the endocytic proteins [20–21]. In this study, we examined the effect of insulin induced Akt activation on albumin endocytosis in proximal tubule epithelial cells. We further investigated how perturbations in insulin signaling lead to alterations in expression of megalin, cubilin in the endocytic pathway in a mouse model of type 1 DM (T1DM). Furthermore we examined whether urinary shedding of cubilin residues occur prior to development of MA in a cohort of patients with T1D to validate its use as a potential biomarker.

## Results

### Insulin treatment induces albumin endocytosis

**Fig 1:**
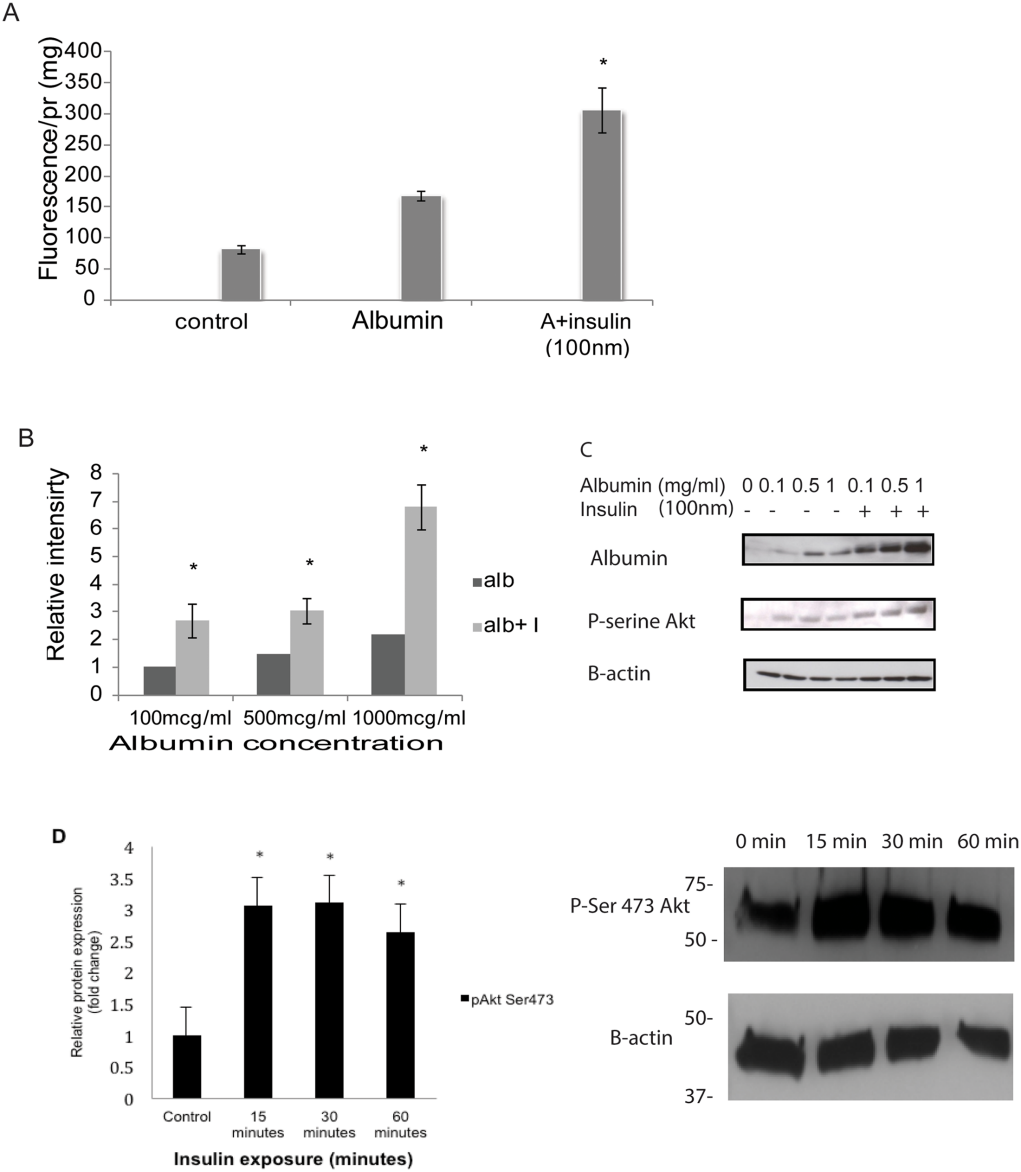
Insulin induces albumin uptake in proximal tubule cells. HKC-8 cells were treated with 100nm of insulin (I9278, Sigma) for 30 minutes followed by FITC-albumin (100μg/ml, A7016, Sigma) for an additional 30 minutes and transferred to 4°C, stripped off membrane bound albumin and lysed. Fluorescence was measured and normalized for the amount of protein. Insulin treatment resulted in an increase in albumin uptake (A). HKC-8 cells were pretreated with insulin (100nm) and incubated with increasing concentrations of albumin. Cell lysates were probed with rat albumin antibody (MP Cappel 55715). Albumin uptake was induced in all concentrations of albumin with insulin treatment in association with an increase in pser473-Akt (Cell Signaling) expression (B, C). Western blotting of cell lysates treated with insulin (100nm) revealed that Akt-pSer473 was upregulated significantly starting at 15 minute of incubation at 30 and 60 minutes (D). *p<0.05.

Confluent monolayer of HKC-8 cells was incubated with insulin (100nm) for 30 minutes and FITC-albumin100 μg/ml for an additional 30 minutes. Cells were washed with PBS^++^, membrane bound albumin is stripped by and lysed. Degree of albumin endocytosis was evaluated by fluorometric measurement of lysed HKC-8 cells and the values are normalized for protein amount. Insulin treatment resulted in a significant increase in albumin endocytosis in the proximal tubule epithelial cells (Fig 1A) (n = 5). To examine if insulin treatment induces albumin endocytosis in higher concentrations of albumin, HKC-8 cells were treated with increasing concentrations of albumin. Western blotting of HKC-8 cell lysates demonstrated that insulin (100nm) treatment causes an increase in albumin endocytosis at higher concentrations of albumin treatment in correlation with phosphorylation of Akt at Ser473 residue (Fig 1B and 1C) (n = 3). Phosphorylation of Akt at 473 serine residue was crucial for its translocation to the plasma membrane to initiate insulin mediated signaling events. Insulin treatment of HKC-8 cells at 15, 30 and 60 minutes induced expression of pSer473 Akt confirming of the link between insulin and activation of Akt in proximal tubule epithelial cells (Fig 1D) n = 5.

### Basolateral insulin treatment causes in an increase in albumin endocytosis

**Fig 2:**
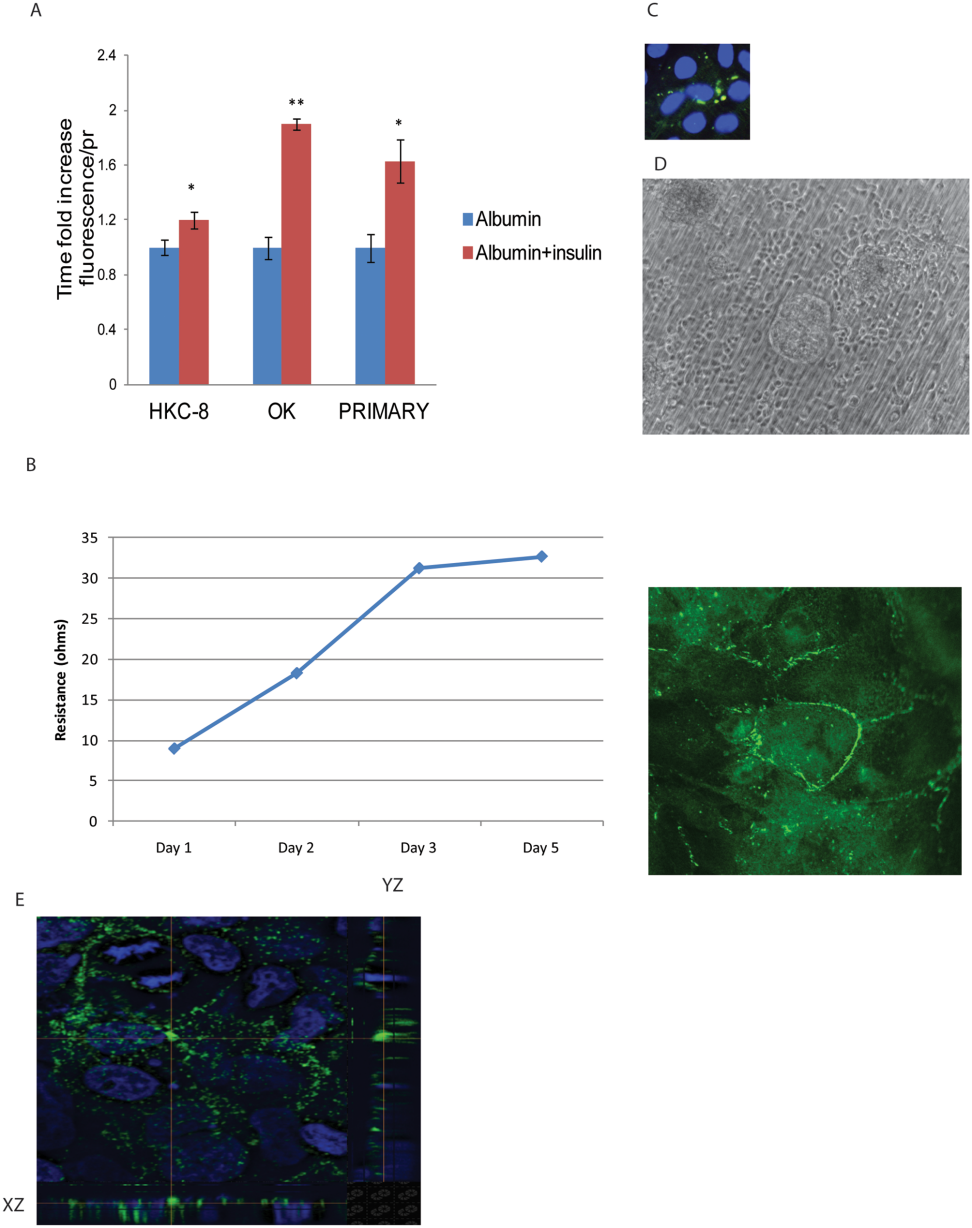
Basolateral insulin treatment induces albumin endocytosis on polarized proximal tubule epithelial cells. HKC-8, OK and primary mouse proximal tubule cells were grown on transwell permeable supports and treated with 100nm insulin basolaterally for 1 hour. FITC-alb (100μg/ml) was added to the apical site ½ hour into insulin treatment. Cells were lysed after PBS^++^ washes. Values were displayed as time-fold increase of albumin uptake in comparison to control cells. Proximal tubule epithelial cells of different origin exhibited an increase in albumin uptake by basolateral insulin treatment (A). Trans Epithelial Electric Resistance (TEER) was measured (ohm) by EVOM epithelial voltohmmeter. Resistance obtained from a well with only media was subtracted from the other wells. Cells reached a stable resistance at 4 days after seeding and expressed tight junction protein ZO1 (B). Localization of e-cadherin at the lateral aspect of the adherence junctions was captured by XY, XZ and YZ confocal section images (E). Primary mouse proximal tubule cells express megalin and form a confluent monolayer 6–8 days after seeding (C, D).

HKC-8 cells seeded on collagen coated polycarbonate transwell membrane formed a polarized monolayer. The resistance across the membrane was measured by EVOM transepithelial electric resistance (TEER). Formation of cell-cell junctions was confirmed by ZO1 staining (Fig 2B). Expression of e-cadherin at the lateral surface of the adherence junctions was demonstrated at the x-y, x-z and y-x planes (Fig 2E). HKC-8 cells are incubated with insulin (100nm) at the luminal chamber for an hour and FITC-albumin (100μg/ml) was added to the apical chamber for the last 30 minutes. Cells were washed, stripped of membrane bound albumin and lysed. Fluorescence was measured and adjusted for protein amount as indicated on the previous figure. The same procedure was repeated for opossum kidney (OK) proximal tubule epithelial cells and primary mouse kidney proximal tubule epithelial cells. Basolateral insulin treatment induced apical albumin uptake in the proximal tubule epithelial cells of different origins (HKC-8, OK cells, primary mouse) confirming the link between insulin signaling and albumin endocytosis (Fig 2A) (n = 6, n = 3, n = 3 respectively). Primary mouse proximal tubule cells displayed cobblestone appearance of epithelial cells by phase contrast microscopy. Primary mouse proximal tubule epithelial cells were stained positive for megalin confirming its origin (Fig 2B and 2C).

### Insulin-induced albumin endocytosis is mediated by Akt

**Fig 3:**
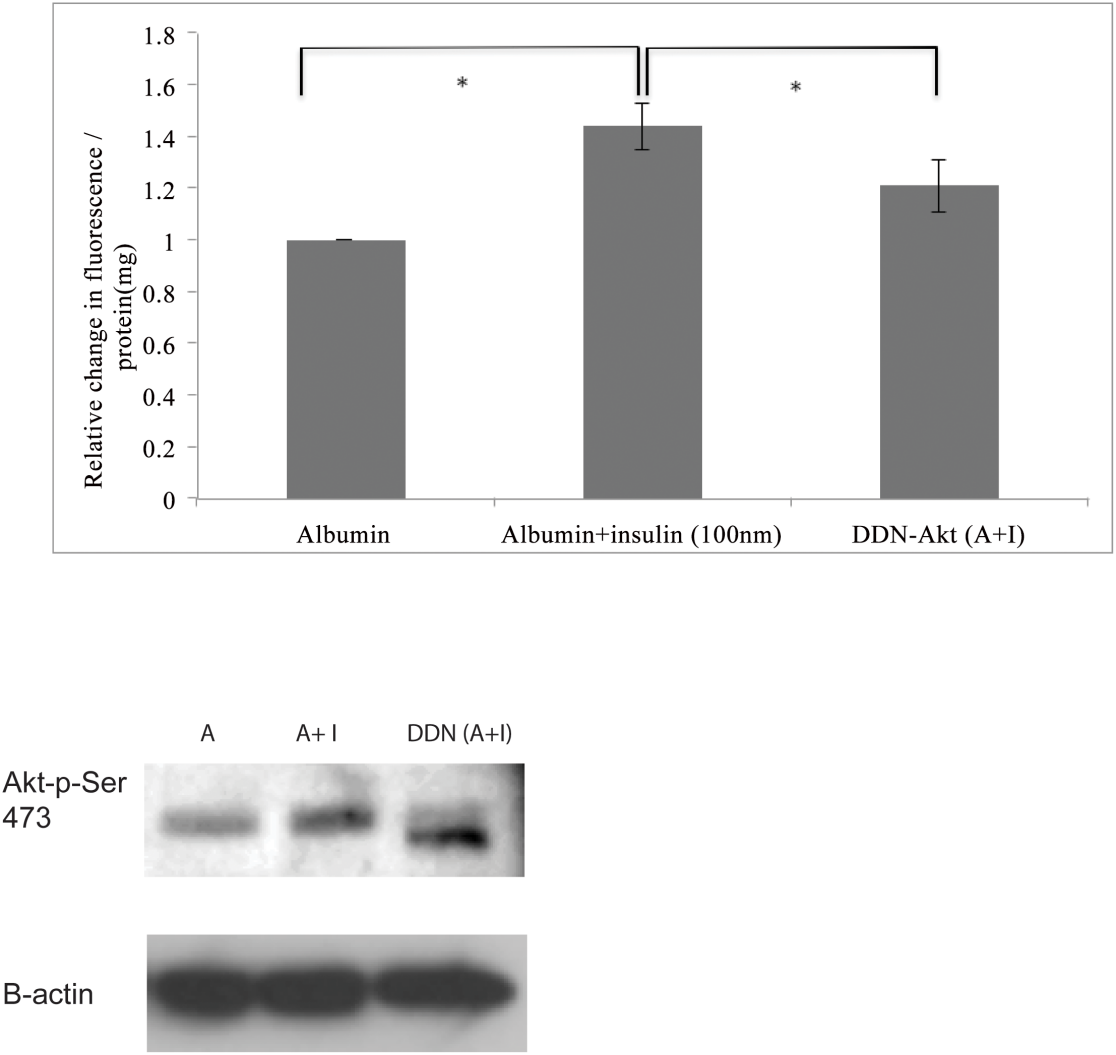
Akt mediates insulin-induced albumin endocytosis. Transfection of HKC-8 cells with a double dominant negative Akt construct resulted in a decrease in insulin induced endocytosis (p<0.05). The comparisons were performed between albumin treated and insulin+albumin treated cells. Insulin treatment resulted in a statistically significant increase in albumin uptake. In order to examine the role of Akt in insulin-induced albumin endocytosis, a comparison of albumin uptake was performed between control cells and cells transfected by DDN Akt plasmid after insulin treatment as depicted in the Fig. Western blot images of the samples revealed that cells transfected with DDN had a lower band with exposure to Akt-pser473 antibody (Cell Signaling) which is consistent with the lack of phosphorylation ability at the pser473-residue.

HKC-8 cells were transfected with a plasmid encoding double dominant Akt (Akt-T308A/S473A) and treated with insulin (100nM) and albumin (100nm). Insulin’s effect on albumin endocytosis was abrogated by inhibition of Akt phosphorylation suggesting that insulin induces albumin endocytosis in the proximal tubule epithelial cells through activation of Akt (Fig 3) (n = 6).

### Insulin treatment resulted in upregulation of Akt phosphorylation sites of AS160

**Fig 4:**
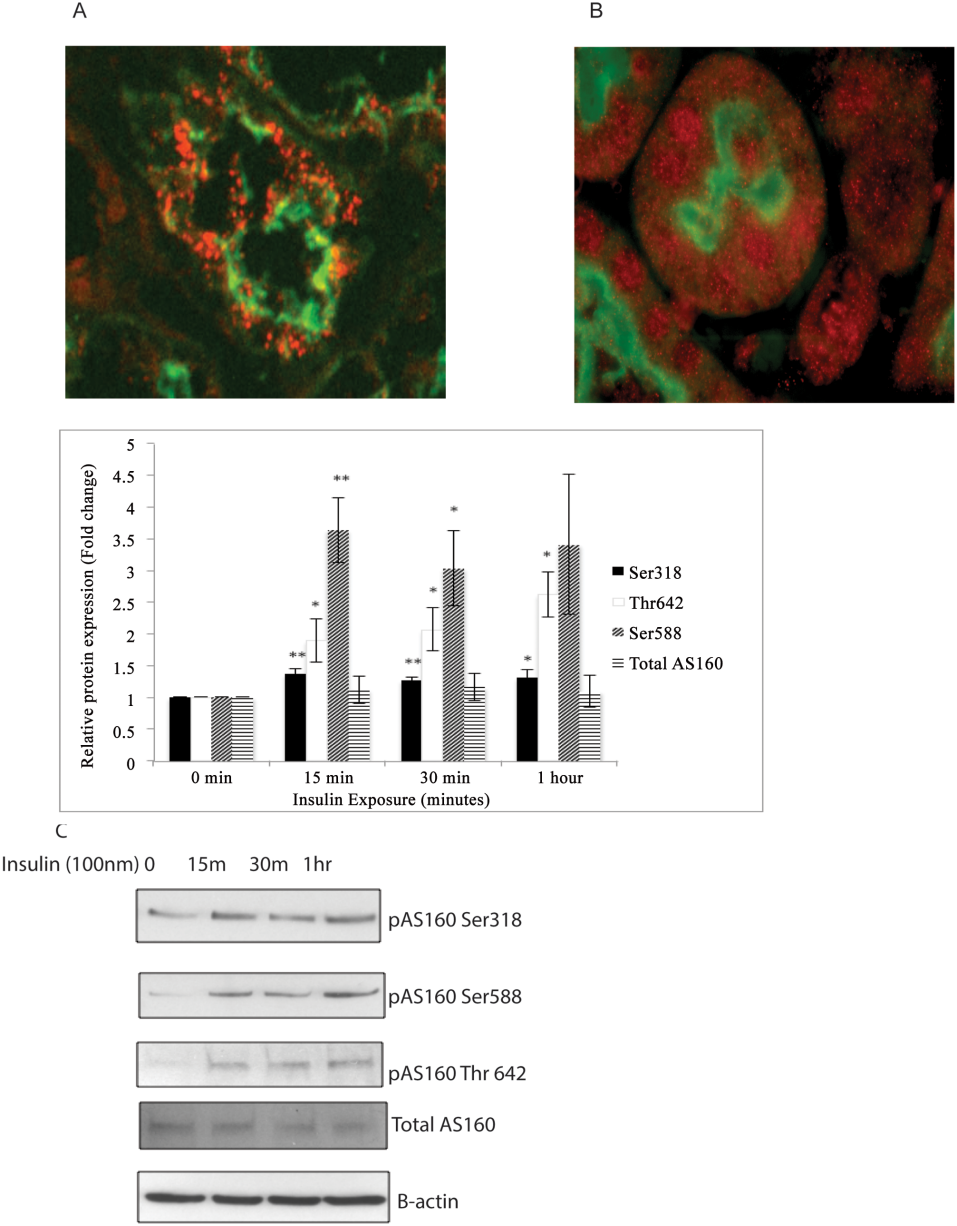
Insulin treatment results in upregulation of Akt phosphorylation sites of AS160. Akt substrate 160 kd (AS160) mediates GLUT4 trafficking in response to insulin in adipose tissue and muscle. The possible physiological effects of AS160 in proximal tubule are unexplored. We first showed that AS160 is expressed in human and mouse proximal tubule epithelial cells (Fig A-B). To explore the effect of insulin induced Akt activation on phosphorylation of AS160, we treated HKC-8 cells with insulin (100nm) for 15, 30 and 60 minutes and investigated expression of Akt phosphorylation sites of AS160. Densitometric measurements were normalized for β-actin and expression of AS160-Ser318, AS160-Ser 588, AS160-Thr 642 and total AS160 at 15, 30 and 60 min of insulin exposure were compared with baseline untreated samples (0 min). Insulin treatment induced phosphorylation of AS160 at Akt phosphorylation sites, S318, S588 and Thr 642 sites in proximal tubule epithelial cells (C). *: p<0.05 ** p<0.01.

Akt substrate 160 kd (AS160) mediates the effect of insulin on GLUT4 trafficking in adipose tissue and muscle. AS160 is involved in aquaporin-2 (AQP2), Na^+^-K^+^ ATPase and distal nephron epithelial sodium channel (ENaC) trafficking in the kidney [22–24]. We explored the possible effect of AS160 as a mediator of albumin endocytosis in proximal tubule epithelial cells. We first showed that AS160 is expressed in human and mouse proximal tubule epithelial cells (Fig 4A and 4B). Then we examined the effect of insulin treatment on phosphorylation of AS160 by Akt. We treated HKC-8 cells with insulin (100nm) for 15, 30 and 60 minutes and examined Akt phosphorylation sites of AS160 in response to insulin. We demonstrated that insulin induces Akt phosphorylation of AS160 at S318, Thr 642 and S588 residues. These results showed for the first time that insulin-Akt-AS160 signaling cascade is operational in proximal tubule epithelial cells (Fig 4C) (n = 5)

### AS160 interacts with megalin-cytoplasmic tail (CT)

**Fig 5:**
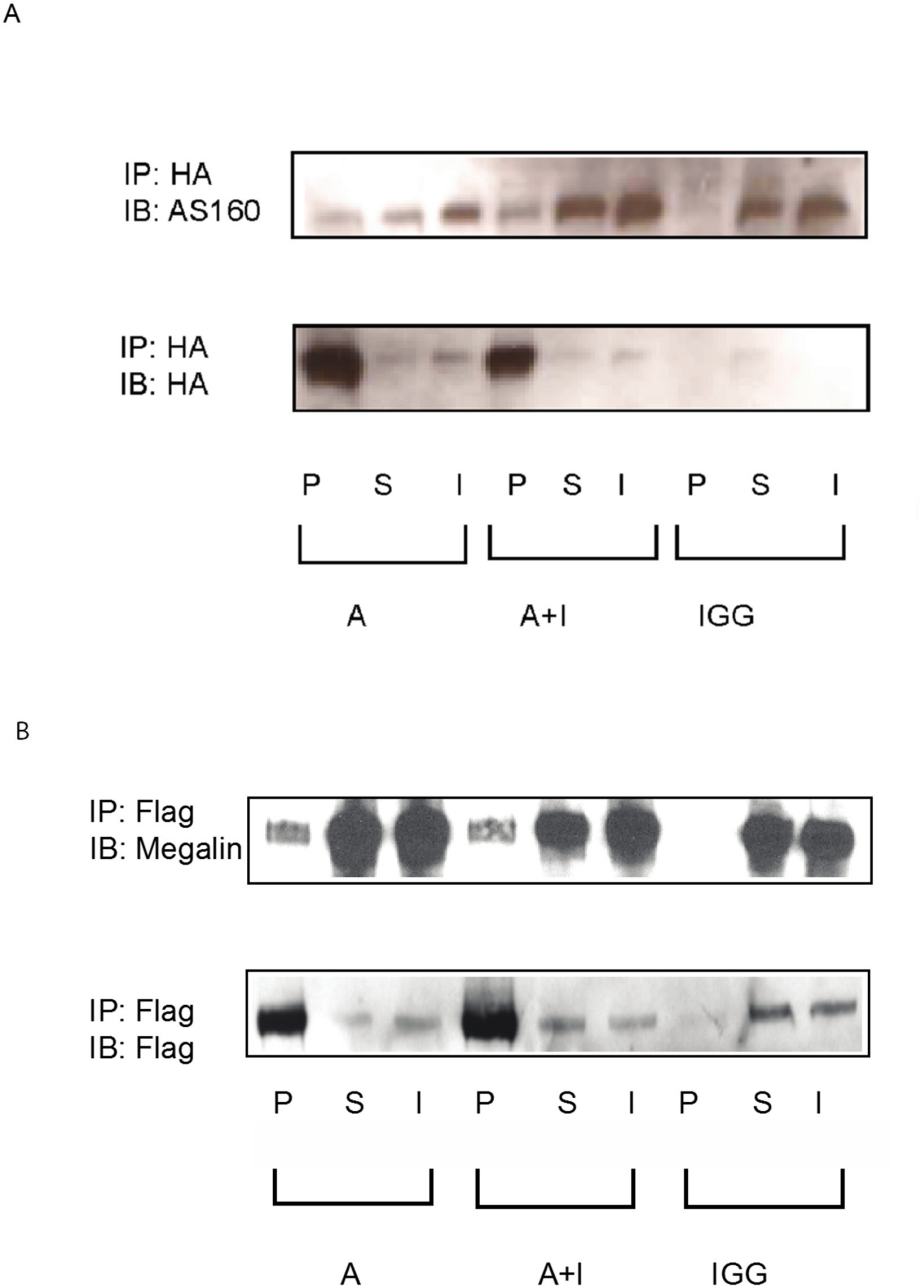
AS160 interacts with megalin. HKC-8 cells were transfected with HA-minimegalin and treated with only albumin (A) and insulin+albumin (A+I). Megalin was pulled down by immunoprecipitation with a HA antibody (Roche) and probed for AS160 (rabbit, Milipore). Mouse IgG is used as negative control. The protein expression in the pellet (P), supernatant (S) and input (I) were displayed. The pellet showed significant AS160 expression where as negative control did not display any protein expression indicating a robust interaction between AS160 and megalin. Insulin treatment caused a modest increase in this interaction. The same blot was stripped and reprobed by a HA antibody to show the efficiency of the pull-down experiments (A). Reciprocal immunoprecipitation was accomplished by transfecting the HKC-8 cells with Flag-AS160 and minimegalin plasmids. AS160 was pulled down by immunoprecipitation with a Flag antibody (Sigma) and the membrane was probed for megalin. It was confirmed that megalin has a strong interaction with AS160. Insulin treatment did not alter the degree of interaction between AS160 and megalin. Pull-down with mouse IgG was utilized as a negative control. The same membrane was stripped and reprobed by a Flag antibody (the image at the lower panel) to examine the efficiency of pull-down experiment (B). Both experiments revealed a strong interaction between megalin and AS160. Megalin and AS160 were successfully pulled down by tag antibodies.

**Fig 6:**
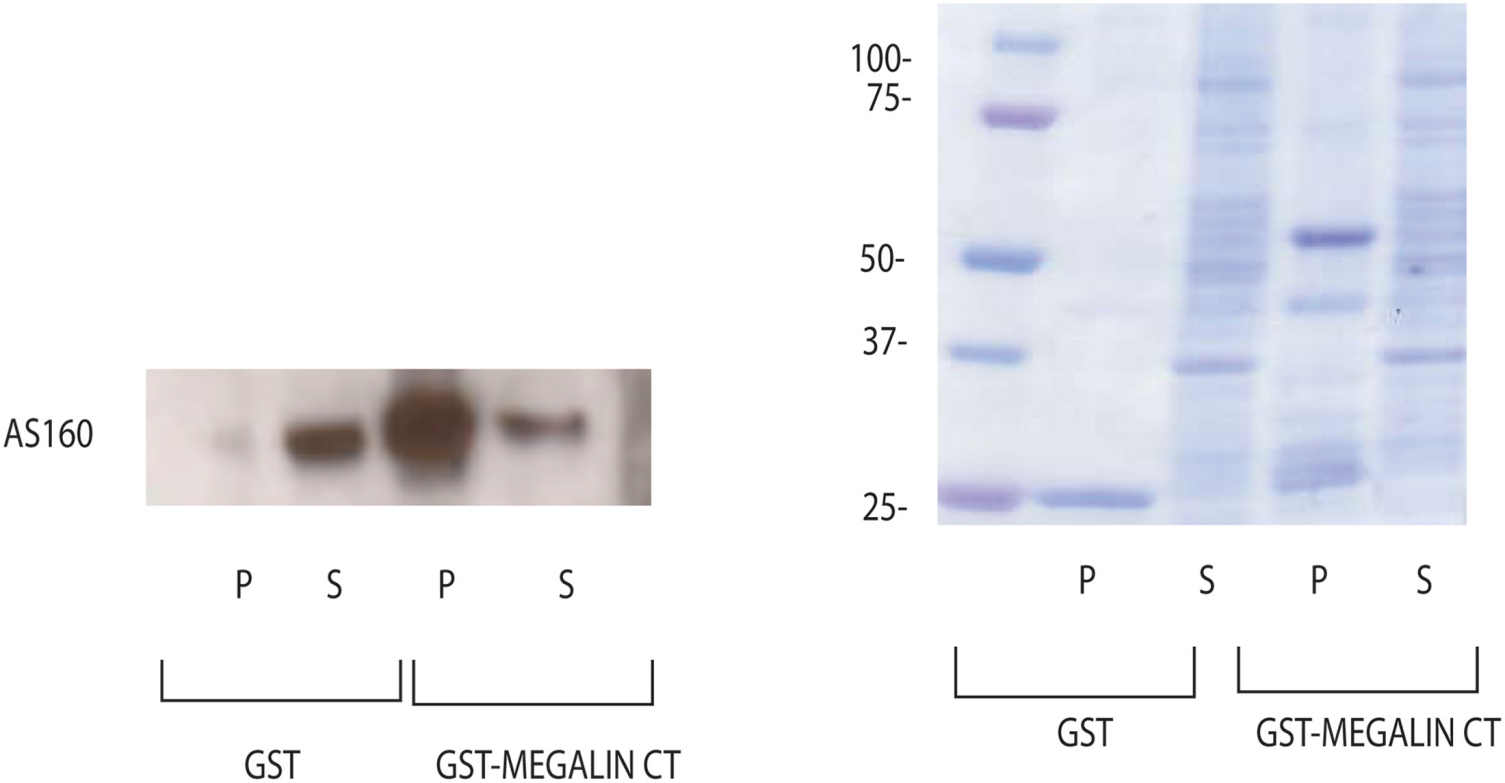
AS160 interacts with cytoplasmic tail of megalin. In order to examine a possible direct interaction between cytoplasmic tail (CT) of megalin and AS160, *in-vitro* pull-down experiments were carried out. Cytoplasmic tail of megalin was cloned and GST-megalin fusion protein was generated as outlined in the methods section [21]. Approximately 100μg of GST, GST-megalin CT immobilized on GSH-Sepharose were incubated with HKC-8 cell lysate. After centrifugation, aliquots corresponding to 1/60 of each supernatant (S) and 1/5 of each washed pellet (P) were resolved by SDS-PAGE and probed for AS160. Pull-down experiments demonstrated a direct interaction between GST-megalin CT and AS160. Only GST was used as a negative control. Coomassie blue staining was utilized to ensure equal loading (B) (n = 3).

AS160 directly associates and affects the intracellular pool of Na^+^, K^+^- ATPase in kidney epithelial cells [22]. We proposed that AS160 interacts with megalin and facilitates its intracellular trafficking. We showed that AS160 interacts with megalin with coimmunoprecipitation experiments (Fig 5A and 5B) (n = 3). In order to examine a direct interaction between cytoplasmic tail of megalin and AS160, we performed GST pull-down experiments. We further demonstrated that AS160 interacts with the cytoplasmic tail of megalin directly by GST pull-down assay (Fig 6) (n = 3).

### Mouse model of T1D introduced by streptozotocin(stz) caused a decrease in megalin, cubilin and AS160 expression

**Fig 7:**
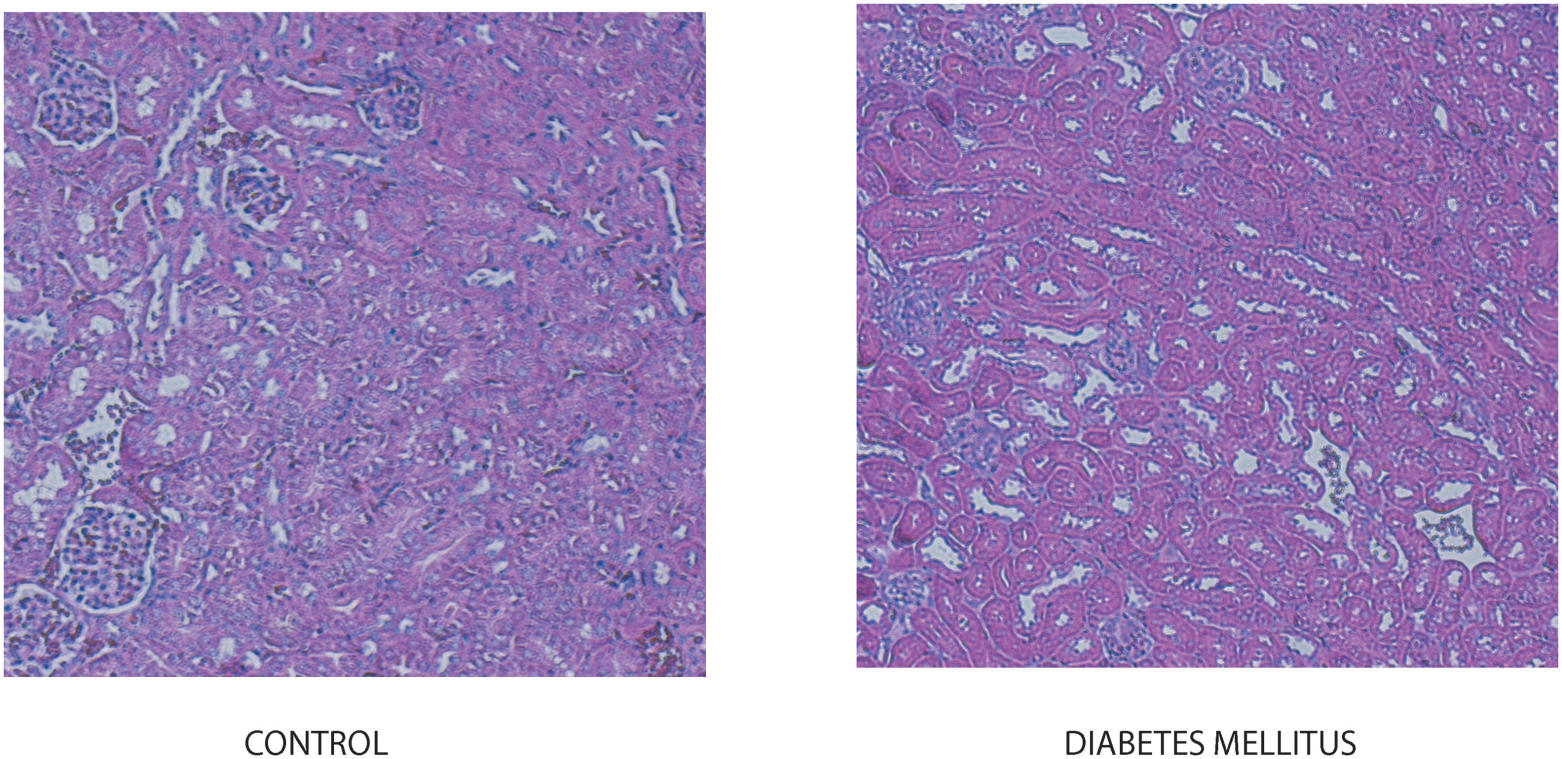
Diabetic mice have normal kidney morphology. Mice were sacrificed and kidneys were removed and stained to examine morphology five weeks after stz injection. Periodic acid-Shiff staining revealed normal glomerular and tubular structure in diabetic mice at 5 weeks after stz injection.

T1D was introduced in mice by low dose stz injections. Examination of the renal morphology by PAS staining did not reveal any significant morphological diabetic alterations in mice five weeks after stz injection (Fig 7). Urinary albumin excretion increased significantly at 5 weeks of stz injection.

**Fig 8:**
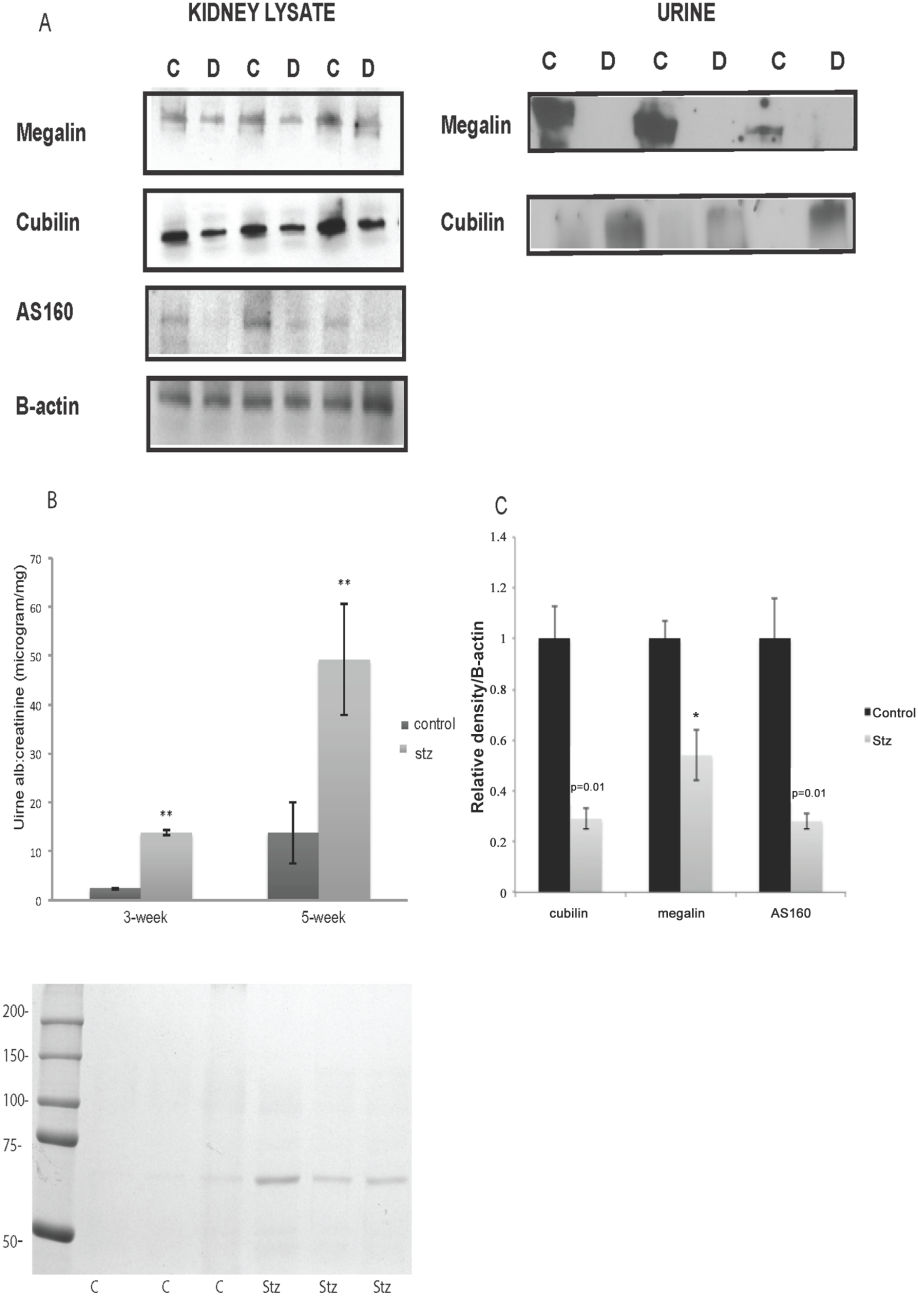
Diabetic mice display a decrease in expression of endocytic proteins associated with alterations in their urinary shedding. Diabetic and control mice kidneys were removed five weeks after stz injection. Western blotting to investigate the expression of albumin receptors megalin and cubilin and downstream Akt substrate was performed. Diabetic mice had downregulation of megalin, cubilin and AS160 expression in their kidney (A). Mice injected with stz to induce T1D and control animals underwent 24hr urine collection in metabolic cages 3 and 5 weeks after stz injection. Urinary proteins were precipitated and examined for megalin and cubilin expression. Urinary shedding of megalin was diminished whereas cubilin shedding was evident in diabetic mice at 3 weeks after stz injection at the time of minimal MA (A). At 3 weeks diabetic mice had minimal MA. A significant increase in MA was observed at 5 weeks after stz injection. Increase in urinary albumin excretion in diabetic mice was confirmed by Coomassie staining (B). Densitometric measurement of western blotting of megalin, cubilin and AS160 adjusted for β-actin revealed a statistically significant decrease in expression of these proteins in stz injected diabetic mice (C) *: p<0.05.

Mice were sacrificed after 5 weeks of stz injection before development of glomerular changes secondary to DN. Mice with T1D had decreased expression of megalin, cubilin and AS160 in their kidneys (Fig 8A). Densitometric measurements adjusted for β-actin reveal a statisticaly significant decrease in expression of megalin, cubilin and AS 160 (Fig 8C). Twenty-four hour urine collections were performed in metabolic cages at 3 and 5 weeks after stz injections. Streptozotocin injections resulted in minimal MA at 3 weeks which was more pronounced at 5 weeks, in concordance with the previous reports (Fig 8B) [25].

Twenty-four hour urine collected at 3 weeks after stz injection displayed a decrease in megalin and an increase in urinary shedding of cubilin preceding development of significant MA (Fig 8A). We postulated that the increase in urinary shedding of cubilin in diabetic mice was a result of mistrafficking of cubilin secondary to decreased membrane expression of megalin and urinary shedding of cubilin occurs prior to development of significant MA.

**Fig 9:**
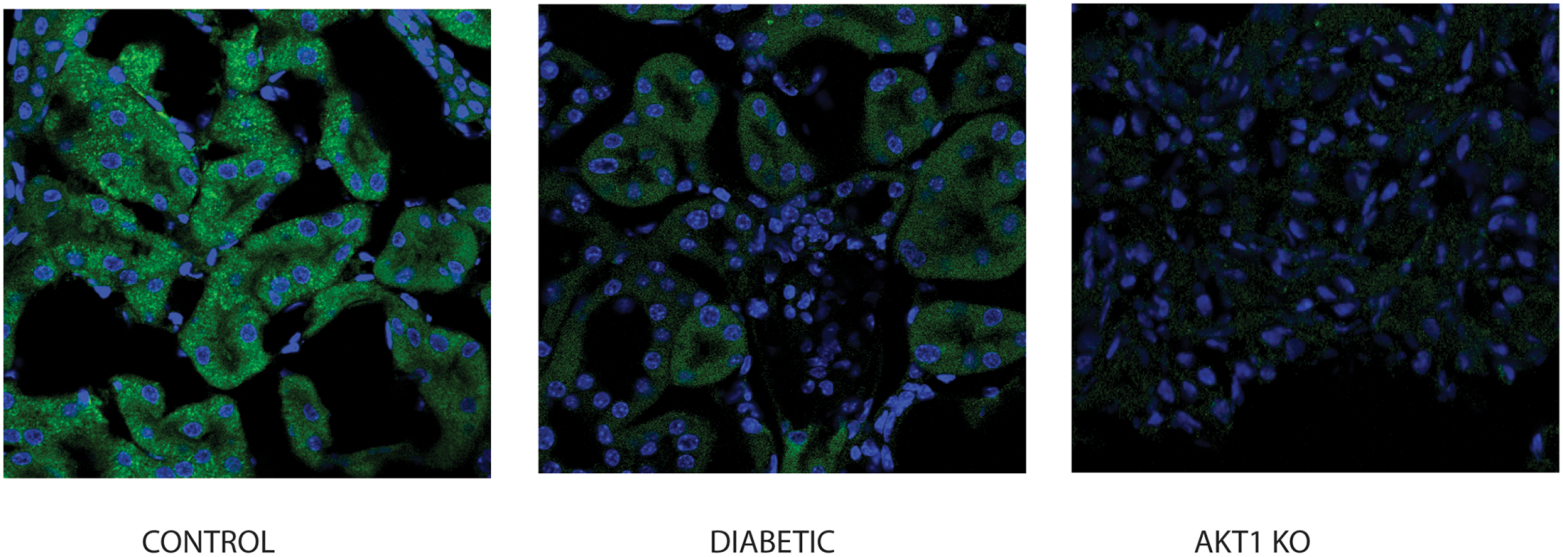
Expression of active Akt is downregulated in diabetic mice kidneys. Immunofluorescence staining of the control, stz injected diabetic mice for Akt-ser473 (Cell Signaling) was performed. Kidneys were double stained with anti-Rabbit FITC. Mouse kidney with knockout of Akt1 (Akt1KO) was utilized as a negative control. Immunofluorescence imaging revealed that phosphorylation of Akt at the 273 serine residue was diminished in diabetic mice kidneys conforming decrease activation of Akt in diabetic animal kidneys before occurrence of significant changes due to T1D.

We next examined expression of Akt-Ser 473 as an upstream mediator of AS160-megalin-cubilin network. Immunofluorescence staining demonstrated a decrease in Akt-Ser 473 expression in proximal tubule epithelial cells of the diabetic mice in comparison to control. Akt1 KO mice kidney was utilized as a negative control (Fig 9).

**Fig 10:**
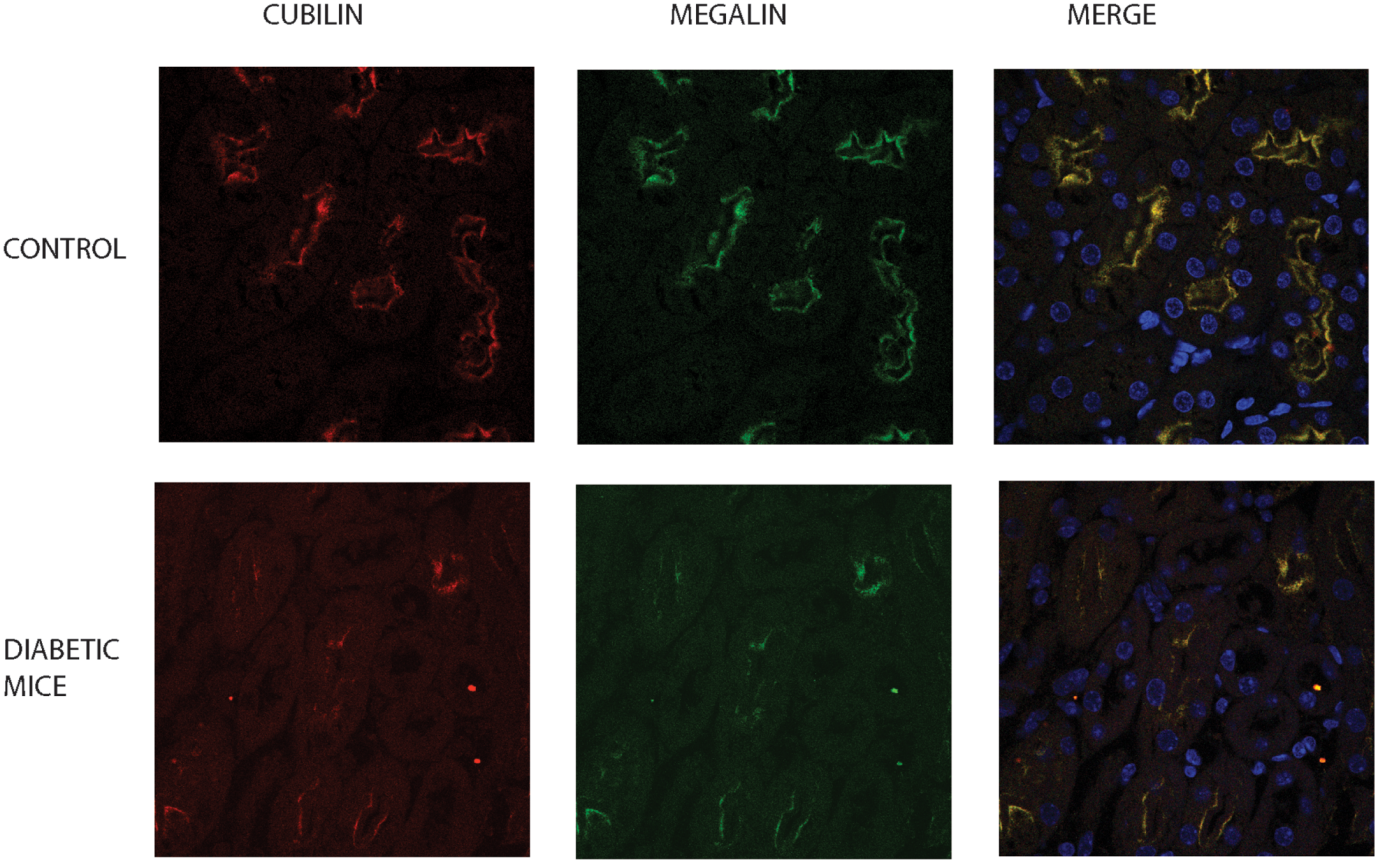
Megalin and cubilin expression is downregulated in diabetic mice kidney. In order to confirm decreased expression of megalin and cubilin in kidneys of diabetic mice, immunofluorescence staining was performed. Kidneys of control and diabetic mice were stained with cubilin and megalin antibodies and double labeled with anti-sheep Alexa-546 and anti-rabbit FITC. Diabetic mice kidneys displayed a decrease in expression of megalin and cubilin in proximal tubule epithelial cells. Hoechst 33342 staining was utilized to delineate the nuclei of the cell.

In order to confirm decreased expression of megalin and cubilin in mice kidney with T1D, we performed indirect immunofluorescence staining. In association with diminished phosphorylation of Akt proximal tubule epithelial cells displayed decreased megalin and cubilin in diabetic mice (Fig 10).

### Patient urines with T1D displayed cubilin shedding prior to development of MA

In an attempt to investigate clinical relevance of urinary receptor shedding, we examined patient urine samples with T1D. We obtained urine samples of patients with T1D who were enrolled to EDC cohort and followed longitudionally for 24 years. Patients who have developed MA were paired with controls who remained normoalbuminuric during their follow-up. Patient pairs were matched for gender and duration of DM. Urine samples were obtained at the time of normoalbuminuria form both groups and the results were normalized for creatinine. Ten pairs of patients (five male and five female) were examined for urinary receptor shedding from overnight samples. After precipitation of urinary proteins as outlined in the methods sections, expression of cubilin was examined by western blotting. Urinary cubilin shedding was detected in nine out of ten patients who eventually developed MA whereas only two normoalbuminuric patients had cubilin shedding. Statistical analysis by Fisher exact test revealed statistically significant urinary cubilin shedding in the group of patients who developed MA during their subsequent follow-up (p<0.01). Urinary cubilin shedding was detected in the MA group 5.23 ± 2.1 years prior to development of MA. Patients with normoalbuminuria maintained their status for a mean of 18.14 ± 2.15 years (10.50–19.47 years). Mean duration of DM in control group was 16.38 ± 4.6 years versus 18.77 ± 6.22 years in the patient group who developed MA (mean ± SD, NS). This data show that cubilin shedding occurs prior to development of MA in patients with DM (5.23±2.1years) and is a potential biomarker to detect early tubular changes due to DM.

## Discussion

The increase in incidence of type1 and type 2 DM is concerning for associated morbidities which will have a negative impact on quality of life of patients with DM. Diabetic nephropathy accounts for the majority of new onset end-stage renal disease in dialysis units. Despite close monitoring of patients for MA and early institution of renin-angiotensin system blockers, not much progress has been made in prevention of DN. Low sensitivity of MA and significant changes in kidney morphology at the time of detection of MA has prompted search efforts to seek more sensitive early biomarkers to detect DN. Diabetic nephropathy is considered a glomerular disease consisting of hemodynamic and metabolic alterations in podocytes, mesangial and endothelial cells which would eventually lead to glomerulosclerosis. Although tubulointerstitial injury correlates better with the renal outcome, there has been very little emphasis on DM related tubular injury in DN. We propose that the molecular cues originating from perturbed insulin signaling in the proximal tubule epithelial cells can be captured in the urine early in DN and understanding the role of insulin mediated downstream signaling pathway in proximal tubule epithelial cells will aid in implementation of early therapeutic interventions. We demonstrated that proximal tubule epithelial cell is not merely a bystander in DN and that tubular changes may precede MA. We showed that proximal tubule is a target for insulin’s actions and insulin signaling initiates a complex cascade of events linking cell signaling to albumin endocytosis. Knocking out insulin receptor in the proximal tubule epithelial cells results in hyperglycemia due to an increase in gluconeogenesis [26]. Insulin signaling in the proximal tubule is linked to Na-HCO3 exchanger and perturbation of this pathway was proposed to be responsible for hypertension observed in metabolic syndrome [27]. We demonstrated that insulin at physiologic concentrations induces albumin endocytosis in proximal tubule epithelial cells through activation of Akt.

Cell signaling events initiated at the plasma membrane are able to govern the uptake and metabolism of ligands by controlling the internalization and recycling of receptors located at the cell membrane. A link between cell signaling and endocytosis has been implicated in different systems. We demonstrated that insulin signaling is tightly connected to albumin endocytosis in the proximal tubule via Akt. Akt is a serine-threonine kinase and implicated in orchestration of cell signaling events including cell survival, proliferation, insulin signaling. Retrieval of albumin from the glomerular filtrate is accomplished by an intricate network of proteins involving megalin-cubilin receptor complex and adaptor protein, Dab2. We previously demonstrated that cell signaling and albumin endocytosis are closely linked through Akt signaling and Akt mediates albumin endocytosis in the proximal tubule through its interactions with the endocytic proteins [20, 21]. Akt also is a pivotal protein in mediating downstream signaling events initiated by insulin. Akt accomplishes this task by phosphorylating key proteins along the pathway. Insulin dependent or independent modulation of Glut4 trafficking by Akt substrate AS 160 was reported in adipose and muscle tissue. AS160 is a GTPase-activating protein (GAP) that promotes hydrolysis of GTP on Rab proteins. Rab-GAP AS160 is implicated in vesicle trafficking, docking and recycling. GAP domain of AS160 is active against Rabs 2A, 8A, 10 and 14 [28]. Among these Rabs, Rab8 was found to be an important mediator of apical trafficking, sorting and polarization [29, 30]. In response to insulin, phosphorylation of AS160 by Akt inhibits its GAP activity and increases GTP-bound form of Rabs promoting translocation of GLUT4 to the plasma membrane in adipocytes and muscle cells [31–33]. In the kidney, AS 160 plays an important role in plasma membrane translocation of aquaporin-2 (AQP2), Na^+^-K^+^ ATPase and distal nephron epithelial sodium channel (ENaC) [22–24]. There are currently no reports of AS160 regulation in the proximal tubule. We demonstrated for the first time that proximal tubule epithelial cells express AS160 and AS160 is phosphorylated at Akt phosphorylation sites Ser318 and Thr 642 in response to insulin. We next investigated how insulin-Akt-AS160 is connected to endocytic machinery in proximal tubule epithelial cells.

**Fig 11:**
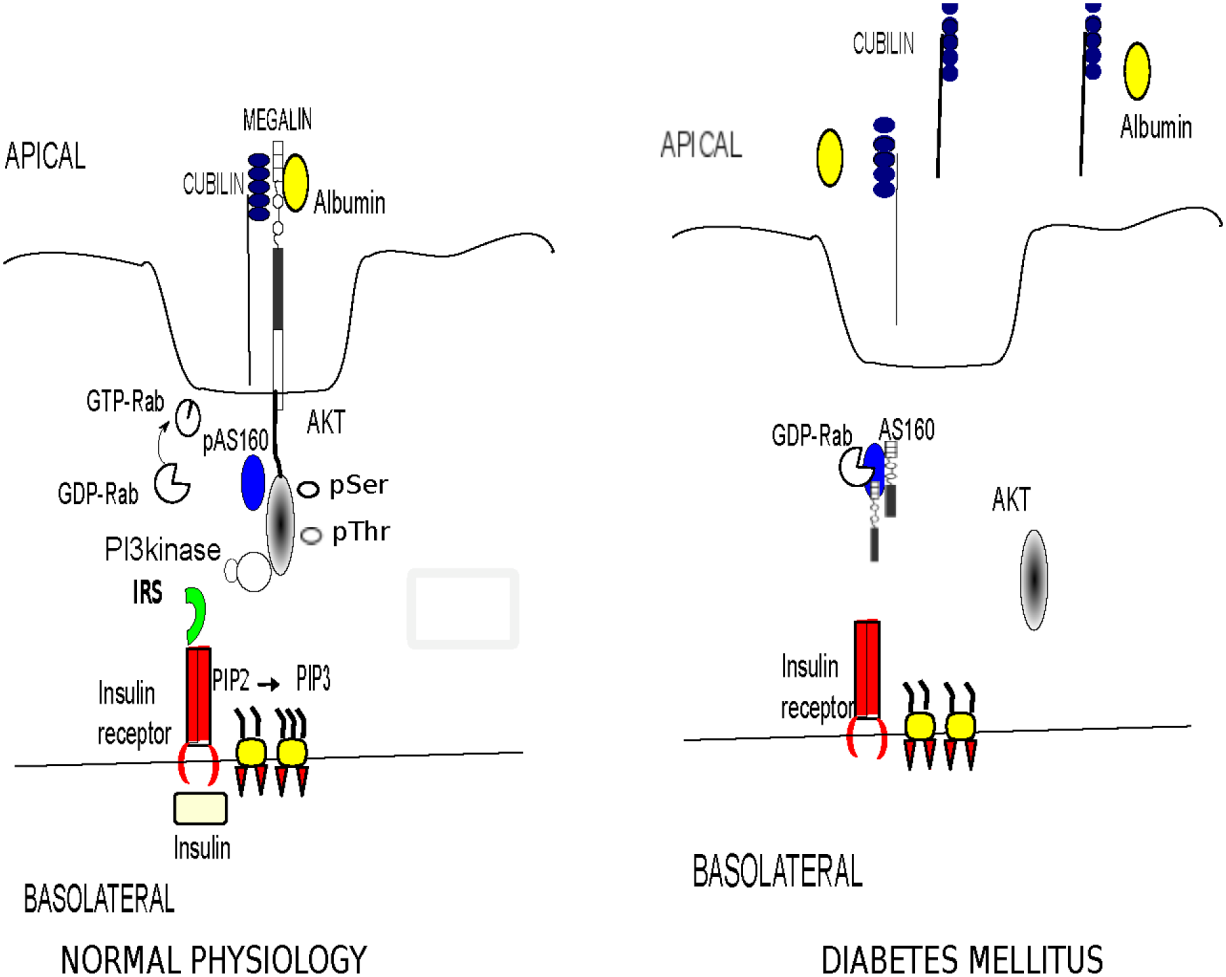
We propose that basolateral binding of insulin to its receptor (IR) is followed by receptor autophosphorylation and activation of tyrosine phosphorylation of insulin receptor substrates (IRS). Binding of IRS to the regulatory subunit of phosphoinositide 3-kinase (PI3K) results in activation of PI3K, which phosphorylates phosphotidylinositol 4, 5 biphosphate (PIP2) on the 3’ position. This complex activates the 3-phosphoinositide dependent protein kinase, PDK-1, resulting in activation of Akt. Active Akt phosphorylates RabGAP AS160 which will increase GTP bound Rabs and recycling of megalin. Increase in megalin turnover will induce apical albumin endocytosis in proximal tubule epithelial cells. In DM this network of insulin induced protein-protein interactions will be perturbed resulting in altered trafficking of megalin-cubilin complex. Decreased membrane expression of megalin will lead to shedding of cubilin in the urinary space.

Megalin and cubilin are responsible for retrieval of 3–5 gram/day of albumin from the glomerular filtrate. Megalin, a member of the LDL receptor family, is a 600-kDa transmembrane protein containing 4600 amino acids. Megalin is essential in apical sorting and endocytosis of ligands particularly albumin, in proximal epithelial cells of the kidney, brain and lungs [34–35]. Megalin also acts as a membrane anchor for the peripheral membrane protein, cubilin, which is a 460-kDa protein with no apparent transmembrane domain and no GPI-anchor. Cubilin deficient mice exhibit a six-fold increase in albumin excretion suggesting its significant role in albumin uptake [36]. Cubilin appears to be essential for albumin uptake whereas megalin drives internalization of cubilin-albumin complex into the cells [36]. We showed that AS160 has an interaction with megalin by *in-vivo* and *in-vitro* protein-protein interaction assays. We propose that insulin induced phosphorylation of AS160 by Akt in the proximal tubule inhibits its GAP activity, shifting the equilibrium of the target Rab to an active GTP-bound form which would increase trafficking and membrane insertion of megalin. Perturbation of insulin signaling in DM will maintain AS160 in unphosphorylated form, which would shift the balance to GDP-bound inactive form of Rabs and result in a decrease in GTP-bound Rabs. The decrease in availability of GTP-bound Rabs will negatively impact recycling and trafficking of megalin and diminish membrane insertion of megalin. Decrease in membrane expression of megalin will inhibit internalization of its partner cubilin leading to its urinary shedding (Proposed mechanism is shown in Fig 11).

In order to test our hypothesis in an animal model of T1D, we utilized a well-established *in-vivo* model of T1D by stz injections to evaluate the link between insulin-Akt-AS160 and albumin endocytosis. In mice chemical induction of T1D by stz caused a decrease in megalin, cubilin and AS160 expression in association with a decrease in pSer473-Akt expression in the proximal tubule epithelial cells. Urinary shedding of megalin was perturbed whereas cubilin shedding was prominent in diabetic animals at the time of minimal microalbuminuria.

Furthermore we demonstrated that human subjects with T1D display urinary cubilin shedding preceding development of MA suggesting that urinary cubilin can be utilized as a biomarker. However although all patients with DM display insulin deficiency or resistance only a certain percentage of the patient population develop MA. It is conceivable to hypothesize that other factors such as genetic variants of cubilin/megalin/Dab2 can play a role in development of receptor shedding and MA.

We showed a novel pathway connecting insulin signaling to albumin endocytosis in proximal tubule epithelial cells. We believe further delineation of delicate protein-protein interactions as part of insulin signaling in proximal tubule will allow us to develop new strategies to diagnose and prevent advanced DN. We propose that early interventions to restore Akt expression in the proximal tubule may result in amelioration of early DN and urinary cubilin shedding can be utilized as an early biomarker in DN.

## Methods

### Cell culture

Human kidney proximal tubule clone-8 (HKC-8) cells (courtesy of Dr. Racussen, John’s Hopkins University) were cultured as published previously [37]. HKC-8 cells are established cell types to study proximal tubule function [38–40]. Medium was changed to serum free media (SFM) overnight before the experiments. Cells were pretreated with 100nm of human insulin for ½ hour and an additional ½ hour during incubation with FITC albumin (100μg/ml, Sigma A9771) or different concentrations of albumin (100μg/ml, 500μg/ml and 1mg/ml, human albumin low-endotoxin, Sigma A5843).

### Western blotting

HKC-8 cells were treated for different durations with insulin (100nm) and lysed in the lysis buffer with protease inhibitors on ice and centrifuged at high speed. Briefly the proteins were separated by SDS-page and probed for Akt-pser 473 (Cell Signaling), total AS160, AS160-Ser318, AS160-Ser-588 and AS160-Thr-642. Protein expression in 15 min, 30 min, 1-hour insulin treated samples were compared with the baseline untreated control (0min) samples.

Fluorometric assay for albumin uptake: Albumin uptake was evaluated by fluorometry and western blotting (WB) as published previously [20, 21]. Cells were washed, transferred to 4°C to stop endocytosis, and washed six times with ice-cold PBS. Any bound but not endocytosed albumin was stripped by ice-cold 0.2 M acetic acid and 2 M NaCl. After multiple washes with PBS, cells were disintegrated by detergent (Triton X-100, 0.1% vol/vol in MOPS solution) and fluorescence was read at 492-nm excitation and 520-nm emission. Fluorescence was normalized for protein after determination of protein amount by Bradford method. The results were expressed as fluorescence/gram of protein.

Immunofluorescence. Three independent trials including 2 separate paired experimental sets (total of 6 samples) were included in the data set. T-test was utilized to compare the normalized fluorescence readings of the groups. The comparisons were performed between albumin treated and insulin+albumin treated cells. In order to examine the role of Akt in insulin-induced albumin endocytosis, albumin uptake in albumin and insulin treated HKC-8 cells with and without DDN-Akt transfection were compared.

HKC-8 cells were seeded on collagen-coated polycarbonate transwell permeable supports (Corning 3413) at 3X10^5^ cells/ cm^2^ [41]. Trans Epithelial Electric Resistance (TEER) was measured (ohm) by EVOM epithelial voltohmmeter. Resistance obtained from a well with only media was subtracted from the other wells. HKC-8 cells, OK and primary mouse proximal tubule cells grown on transwell support were treated with 100nm insulin basolaterally for 1 hour. FITC-alb (100μg/ml) was added to the apical site ½ hour into insulin treatment. Cells were lysed after PBS^++^ washes. Values were displayed as time-fold increase of albumin uptake in comparison to control cells. Primary mouse proximal tubule cells were isolated from C57BL/6 mice by collagenase treatment and passing the cell suspension through different size sieves [42]. Primary proximal tubule cells formed a confluent monolayer 5–6 days after seeding. The origin of the cells was confirmed by megalin staining.

### Glutathione S-transferase (GST) fusion pull-down experiments

Cytoplasmic tail of megalin was cloned by PCR using LRP-mini megalin cDNA as template. All constructs were verified by automated sequencing. Expression of inserts cloned into a pGEX-4T-1 vector under the control of the isopropyl-β-D-thiogalactoside (IPTG) inducible tac promoter was performed for production of GST-megalin CT. All constructs were verified by automated sequencing. GST and GST fusion protein containing megalin-CT construct was produced in *Escherichia coli* B21 cells. Bacteria were induced by shifting log phase cultures at OD_600_ = 0.6. After the initial growth, bacteria were induced by adding 100μm of IPTG with constant shaking at room temperature for 3 hours and centrifuged at 15,000 X g at 4°C for 15 min and stored at -80°C. Bacteria were lysed on ice with a sonicator in 50mM Tris-HCl, pH7.5, 300mM NaCl, 0.2%(w/v) Triton X-100, 10mM β mercaptoethanol. Insoluble material was removed by centrifugation at 23,700Xgmax at 4°C. GST fusion proteins were immobilized on glutathione-sepharose. After extensive washing in PBS, GST fusions were eluted in assay buffer (25mM Tris-HCl, pH 8, 200mM NaCl, 10mM glutathione, 5mM DTT) on ice and dialyzed into PBS before use in binding assays [20, 21].

#### Binding assays

Dialyzed GST-fusion proteins were bound to glutathione-Sepharose and then mixed with clarified HKC-8 cell lysate to give a final concentration of ~7.5 mg/ml in 300 μl of total volume. After incubation at 4°C for 60 min, the beads were separated by centrifugation, and aliquots corresponding to 1/60 of each supernatant (S) and one fifth of each washed pellet (P) was resolved by SDS-PAGE and either stained with Coomassie blue or transferred to nitrocellulose membrane. The blot was probed with AS160 antibody (Milipore) [20, 21].

### Preparation of cell lysates

*C*ells were washed with PBS^++^ and incubated 30 minutes in ice cold lysis buffer (20 nM Hepes/pH 7.4, 2 mM EGTA, 1%Triton X-10/400 μM PMSF/50 mM NaF/2μM microcystin LR/2Xcomplete protease inhibitor (Roche) 10ng/μl leupeptin/10ng/μl aprotinin/4ng/μl elastatinal/2.5mM, phenanthroline/100μM L-1ltosylamido-2phenylethyl chloromethyl ketone (tpck) and sonicated for 10s 3 times and cleared by centrifugation at 4°C for 10 minutes at 15,000 x g.

### Preparation of the kidney lysates

Control and stz treated mice were sacrificed after five weeks of stz injection. Kidney lysates were prepared after homogenization with Dounce homogenizer in lysis buffer with protease inhibitors. After high-speed centrifugation proteins were separated by SDS page and probed for megalin, cubilin and AS160.

### Plasmids

To investigate a possible interaction between megalin and AS160, HKC-8 cells were transfected with HA-tagged mini megalin (chimeric plasmid encoding LRP minireceptor consisting the fourth ligand-binding ectodomain followed by transmembrane domain of LRP and 209 amino acids of the megalin tail-mLRP/LRPTmMegT, courtesy of Goujun Bu) [43].

Cell lysates were immunoprecipitated with HA mouse antibody (Covance HA.11 Clone 16B12 Monoclonal Antibody) and probed for AS160 (Milipore). The same membrane was stripped and reprobed with the antibody against HA tag to demonstrate the efficiency of transfection and immunoprecipitation. Supernatant (S) and 1/60 of the cell lysate (input-I) were used to examine the degree of the interaction. For reciprocal immunoprecipitation, HKC-8 cells were double transfected by Flag AS160 (courtesy of G. Leinhard) and mini megalin plasmid.

A plasmid encoding dominant negative Akt (Akt-T308A/S473A, courtesy of Rojanasakul Y, West Virginia University) was utilized to inhibit PKB/Akt expression [44]. The cDNA was mixed with Lipofectamine-2000 (Invitrogen) in Opti-MEM reduced serum media (Invitrogen) and incubated overnight. Media was changed to F12: DMEM with 5% FBS the following day. Experiments were performed 48 hours after transfection. Cells were maintained in serum free media (SFM) for 16 hours before the experiments

### Immunofluorescence

To localize AS160 paraffin blocked healthy human kidney (patient with Wilm’s tumor who underwent nephrectomy) and mouse kidney (C57BL/6J) sections were deparaffinized and rehydrated with xylene and decreasing concentrations of ethanol. After fixation with 4% paraformaldehyde PFA, kidney sections were permeabilized with 0.1% Triton in PBS and stained with rabbit AS160 antibody (courtesy of J. Loffing, University of Zurich, Switzerland) and mouse AS160 antibody (Sigma).

HKC-8 cells grown on transwell inserts were stained for cell to cell junction zonula occludens (ZO-1) protein and E-cadherin, a transmembrane protein that is necessary to maintain adherens junctions of polarized epithelial cells by immunofluorescence 4 days after seeding. In brief cells were fixed by 4% PFA, washed with PBS^++^ and blocked in 1%BSA+ 5% goat serum. Membranes were incubated with ZO1 (Abcam) and E-cadherin (Santa Cruz) antibodies overnight at 4°C and cut and visualized by NikonA1 invert confocal microscope after incubation with Alexa 488 labeled secondary antibody.

For megalin and cubilin staining of kidney sections of stz mice the same procedure was followed without permeabilization. Kidney sections were stained with rabbit megalin (courtesy of D. Biemesderfer) and sheep cubilin antibody (R&D) overnight and exposed with fluorescence labeled secondary antibodies for an hour. Images were captured by Olympus Fluoview 1000 confocal microscopy.

### Animal experiments

Animal protocol related to induction of T1D in mice including intraperitoneal streptozocin, urine collections and kidney harvesting was approved by University of Pittsburgh IACUC. T1DM was induced to C57BL/6J mice by low-dose intraperitoneal stz (50mg/kg) injections for five consecutive days (Jackson Laboratories). Mice with fasting blood glucose levels >300mg/dl was deemed to have developed T1D. Urine collection was performed for 24-hours in metabolic cages at 3 and 5 weeks after development of T1D. Animals were sacrificed at 5 weeks by CO2 and vital organ removal. Urine albumin and creatinine was measured by Albumin (Human) AssayMax ELISA Kit and R&D creatinine kit.

### Patient population

We analyzed urine samples of the patients with T1D, who were followed longitudinally and have developed persistent MA in the cohort of The Pittsburgh Epidemiology of Diabetes Complications (EDC) study for urinary cubilin shedding prior to development of MA to assess the predictive role of urinary receptor shedding. We used already obtained patient urine samples that were collected within the EDC cohort (consents forms from patients were collected at the time of enrollment) therefore a separate consent was not obtained for utilization of the urine samples. IRB approval from University of Pittsburgh was obtained for this study and the need for written consent from the participants was not waived. The NIH funded EDC cohort includes 658 patients with type I diabetes diagnosed between 1950–1980, at Children’s Hospital of Pittsburgh. Mean age of patients was 28 (8–48) with an average follow-up of 24 years. The patients were evaluated at baseline and biennially with estimated GFR calculation and three separate urine collections including a 24-hour, overnight and a urine collection during clinic visits. In EDC study MA was described as albumin excretion rate (AER) 20–300 μg/min on at least 2 of 3 timed urine samples. Patients who developed MA on subsequent follow-up and maintained normoalbuminuric status were paired based on gender and duration of DM. In the EDC study 201 patients have developed MA. Two hundred seventy seven patients maintained normoalbuminuria status throughout the follow-up. Urinary cubilin shedding was examined after precipitation of urinary proteins as described below. Overnight urine samples were utilized.

### Precipitation of the urinary proteins

Urine samples were thawed and mixed with protease inhibitors immediately and centrifuged at 1000 X g for five minutes to remove cell debris and nuclei. Urine volumes were normalized for creatinine. Urine proteins were precipitated with methanol: acetone to preserve the high molecular weight proteins and separated by SDS-PAGE high gradient gel to examine urinary shedding of megalin and cubilin [45].

### Statistical analysis

Densitometric measurement comparisons of western blots were performed by student’s sample t-test by SPSS statistical program. The program performed a two-tailed student’s t-test given a null hypothesis that the two means were equal. A p value of < 0.05 was considered significant.

Fisher’s exact test was used to compare urinary shedding of cubilin between patient populations.
